# Economic impact of industry-sponsored clinical trials in inflammatory bowel diseases: Results from the national institute of gastroenterology “Saverio de Bellis”

**DOI:** 10.3389/fphar.2022.1027760

**Published:** 2022-11-22

**Authors:** Maurizio Gaetano Polignano, Giuseppe Pasculli, Pietro Trisolini, Michele Albino Di Lorenzo, Giuseppe Dalfino, Gianluigi Giannelli

**Affiliations:** ^1^ Scientific Management Department, IRCCS National Institute of Research “Saverio De Bellis”, Bari, Italy; ^2^ Department of Computer, Control, and Management Engineering Antonio Ruberti (DIAG) Sapienza University, Rome, Italy; ^3^ IRCCS National Institute of Research “Saverio De Bellis”, Bari, Italy; ^4^ Economic Department, IRCCS National Institute of Research “Saverio De Bellis”, Bari, Italy

**Keywords:** clinical research, economic evaluation, cost avoidance, leverage effect, gastroenterology

## Abstract

**Introduction:** The majority of the money spent on possible new medications’ clinical trials is accounted for by the innovative pharmaceutical sector, which also stimulates the economy of a nation. The objective of this study was to evaluate the impact of pharmaceutical industry-sponsored clinical trials (ISCTs) in inflammatory bowel diseases (IBDs) towards the national health service (NHS) in terms of avoided costs and leverage effect.

**Methodology:** The research was conducted at National Institute of Gastroenterology, “Saverio De Bellis”, Castellana Grotte (Apulia, Italy) collecting data from profit ISCTs of pharmaceutical products conducted over the time period 2018-2020 with focus on inflammatory bowel diseases. After the quantification of health services and drug costs from the latter studies, avoided costs and leverage effects were then estimated.

**Results:** The results on the avoided costs for healthcare facilities deriving from the conduct of clinical studies show that, in relation to the sample of five drug companies participating in our 2018-2020 analysis, out of a total of 235,102.46 €, identified as direct investment, 628,158.21 € of avoided costs for the NHS were measured, with an additional saving (leverage effect) for the NHS of 3.67 € for each € invested by the companies promoting clinical trials.

**Conclusion:** Conducting profit clinical trials has practical benefits and a favourable macroeconomic impact that, by completing its limited resources, helps to sustain one country NHS thanks to the avoided costs while also contributing to locational and industrial policy while guaranteeing novel therapeutics and health services for the patients enrolled.

## Introduction

Clinical trials hasten the implementation of new procedures and products into routine clinical practice. They are also the foundation for distinguishing effective and life-enhancing medical treatments from ineffective medical treatments. Planning, executing, and evaluating clinical trials in accordance with international standards necessitates significant resources, infrastructure, and the availability of qualified personnel. It is worth noting that industry sponsorship entails sponsors providing free investigational pharmaceutical products, covering the costs of study-specific diagnostics and other treatments, and compensating for thematic and administrative work.

In light of this, there is an increasing interest towards measuring the contribution of overall value of clinical trials to the national economic system of a country.

In Italy, 672 new clinical trials were approved in 2019, equal to 23% of those approved in the European Union. (Angerame et al., 2020).

The economic value of clinical trials can be expressed by several indirect and direct factors.

With regards to indirect factors, these include induced and positive economic effects of investments towards providers of clinical research services such as Contract Research Organizations (CROs), laboratories, diagnostics, couriers, and others.

In terms of direct factors, the overall economic investments of public and private subjects have been quantified being over 750 Million € per year and, on average, 92% of direct funding comes from pharmaceutical company funding for profit studies. (FADOI, 2019).

In particular, recent research focused on the qualitative description of the value of clinical research for the socio-economic system or on the estimate of the economic value of the research, with a focus on the costs avoided by the National Health Service (NHS) thanks to the drugs provided free of charge by the companies promoting clinical trials (so-called “Averted” or “Avoided” costs).

The most immediate avoided cost is due to the free provision of experimental and control drugs administered to patients enrolled in clinical trials, the costs of which are entirely borne by the sponsoring companies. To these must be also added all the numerous diagnostic services and laboratory analyses that are performed throughout the clinical studies. Both these drugs and these services, if patients were not enrolled in a clinical trial, would have been provided by the NHS, hence bearing the relative costs.

Recent studies indicate that every € invested in clinical trials by the pharmaceutical industry generates between 1.95 and 2.50 € of added value for the economy as a whole (Walter et al., 2020), (Grueber, 2015) along positive effects on employment, with the use of highly specialised professional profiles, both medical and managerial. Indeed, the aforementioned study estimated the “employment multiplier” effect of clinical research being 1.66.

Furthermore, it should be noted that in some countries such savings have long been studied as a tool for reducing financial pressure on NHS, as evidenced for example by a Koçkaya et al. (2015), which highlighted how the Turkish NHS was able to free up resources amounting to 31 Million $ in the period 2006-2010 thanks to the free supply of drugs in clinical trials provided by sponsors.

In another study conducted in Taiwan (Shen et al., 2011), the drug cost avoidance from sponsored clinical trials was calculated by year, trial, patient, therapeutic area, and phase. Three-quarters of the cost avoidance in medication expenditures was calculated from 194 funded clinical studies and amounted, just for the year 2008, to around 11.2 Million $ USD. The average cost avoidance value was 58,000 $ each trial year, or 3,900 $ per participant year. Early-phase trials and phase III trials accounted for 25% and 56% of all trials, respectively, whereas they accounted for 32% and 49% of total costs averted, respectively. The most often conducted and greatest cost-avoiding studies were those for antineoplastic drugs, particularly targeted treatment, which accounted for 85% of overall anti-cancer trial cost avoidance.

In a recent United Kingdom (UK) report by the National Institute of Health Research (NIHR) (https://www.nihr.ac.uk/news/new-report-highlights-how-nihr-support-for-clinical-research-benefits-the-uk-economy-and-nhs/22489#:∼:text=Put%20simply%2C%20clinical%20research%20benefits,of%20new%20drugs%20and%20treatments.%E2%80%9D[Not Available in CrossRef, 2248), it was also highlighted that over the 3-year period (financial years) 2016-2019, on average, NHS providers in England earned an estimated 9,200 £ for each patient recruited into a commercial trial sponsored by the NIHR, hence saving an estimated 5,800 £ per patient (where trial drugs replaced the standard treatment).

The entire expected revenue for the NHS from commercial clinical trials was estimated being around 355 Million £, with a total estimated cost savings of 28.6 million £ (where trial drugs were used in place of standard).

The issue in Italy has been addressed by different studies, all of which have highlighted savings from the conduct of clinical trials. Nonetheless, all studies have so far been conducted on a non-extensive sample of clinical studies (up to a maximum of 37), focused on the onco-haematological area and provided by a small number of companies or health facilities, or on single wards. The previous research on the topic of avoided/averted costs (Cicchetti et al., 2020), analysed the data provided by the Gemelli Polyclinic (Rome, Italy) and the Giovanni XXIII Hospitals (Bergamo, Italy): out of 18 and 22 studies in the oncology area respectively, showed an average leverage effect of avoided costs of 2.2. This means that for every € invested in clinical trials and disbursed by sponsoring companies to health facilities, the NHS saved over 2 €. Based on these values, a total potential saving of approximately 400 Million € has been projected for the NHS in onco-haematology (Angerame et al., 2020).

This methodology has the advantage of producing an indicator that makes the avoided cost-analyses carried out in different contexts comparable.

This study focused on evaluating the avoided costs and leverage effect in clinical pharmacological studies on inflammatory bowel disease (IBD). To our knowledge, no previous studies have been previously conducted in Italy focusing on gastrointestinal diseases/IBD as this present study. IBD is a group of inflammatory conditions of the colon and small intestine, Crohn’s disease and ulcerative colitis being the principal types (Talley, 2018). Crohn’s disease affects the small intestine and large intestine, as well as the mouth, oesophagus, stomach, and the anus, whereas ulcerative colitis primarily affects the colon and the rectum (Baumgart and Carding, 2007).

The amount of the added value of deriving from clinical trials activities in Italy has not been quantified exactly. Hence the multiplier of the overall value generated by clinical research deserves to be further investigated in the Italian context.

This study, carried out with the collaboration of a pool of five supporting pharmaceutical companies, aims to consolidate a model for estimating the direct investment of the companies and the costs avoided thanks to the drugs provided free of charge by the companies, together with the measurement of the leverage effect generated by such investments as a multiplier of benefits for the NHS as a whole with particular focus on gastrointestinal diseases which represents a novel application in Italy.

### Study aims

The aim of this study was to evaluate the economic benefit of ISCTs in IBD towards the NHS in terms of global avoided costs and by estimating its leverage effect.

## Materials and methods

### Database creation

The research was conducted at National Institute of Gastroenterology “Saverio De Bellis”, Castellana Grotte (Apulia, Italy) collecting data from profit industry-sponsored clinical-trials (ISCTs) of pharmaceutical products conducted over the time period 2018-2020.

Data was collected from various sources of data, accounting for 217 pharmaceutical treatments from 41 patients over 11 profit studies sponsored by five major pharmaceutical companies (Table 1).

**TABLE 1:** List and description of profit studies conducted (data sources).

Trial name	Sponsor	Year
GS-US-419-3895 - Combined Phase 3, Double-blind, Randomized, Placebo-Controlled Studies Evaluating the Efficacy and Safety of Filgotinib in the Induction and Maintenance of Remission in Subjects with Moderately to Severely Active Crohn’s Disease	GILEAD SCIENCES	2018
GS-US-418-3898 - Combined Phase 2b/3, Double-Blind, Randomized, Placebo-Controlled Studies Evaluating the Efficacy and Safety of Filgotinib in the Induction and Maintenance of Remission in Subjects with Moderately to Severely Active Ulcerative Colitis	GILEAD SCIENCES	2018
GS-US-418-3899 - A Long Term Extension Study to Evaluate the Safety of Filgotinib in Subjects with Ulcerative Colitis	GILEAD SCIENCES	2018
Phase 2A, double-blind, randomized, placebo-controlled, parallel group study to evaluate the efficacy and safety of PF-06651600 and PF-06700841 administered orally as open-label induction and extension treatment in affected subjects moderate to severe Crohn’s disease (PIZZICATO B7981007)	PFIZER	2019
Phase 2A, randomized, double-blind, placebo-controlled, parallel-group study to evaluate the efficacy and safety of oral PF 06651600 and PF-06700841 as open-label induction and extension treatment in subjects with colitis moderate to severe ulcerative (VIBRATO B7981005)	PFIZER	2019
A phase 2b, Multicenter, Randomized, double-blind, placebo-controlled Dose-ranging study to evaluate the efficacy, safety, and pharmacokinetics of PF-06480605 in Adult participants with moderate to severe ulcerative colitis (TUSCANY 2- B7541007)	PFIZER	2020
APD334-301: A Phase 3, Randomized, Double-Blind, Placebo-Controlled, 52-Week Study to Assess the Efficacy and Safety of Etrasimod in Subjects with Moderately to Severely Active Ulcerative Colitis (STUDIO ELEVATE 52)	ARENA PHARMACEUTICALS	2020
RPC-3201 Induction Study #1 - A Phase 3, Multicenter, Randomized, Double-Blind, Placebo-Controlled Induction Therapy for Moderately to Severely Active Crohn’s Disease	CELGENE	2019
APD334-302:A Phase 3, Randomized, Double-Blind, Placebo-Controlled 12-Week Study to Assess the Efficacy and Safety of Etrasimod in Subjects with Moderately to Severely Active Ulcerative Colitis (STUDIO ELEVATE 12)	ARENA PHARMACEUTICALS	2020
APD334-303: An Open-Label Extension Study of Etrasimod in Subjects with Moderately to Severely Active Ulcerative Colitis	ARENA PHARMACEUTICALS	2020
SATISFACTION_Mannitol 03-2018 study: “Efficacy and safety of mannitol in intestinal preparation: evaluation of the adequacy and presence of intestinal hydrogen and methane levels during elective colonoscopy after administration of mannitol or standard fractional administration of 2 L of polyethylene glycol and ascorbate solution - Phase II/III, international, multicentre, randomized, parallel group, blinded endoscopist, dose definition/non-inferiority study	NTC	2020

### Costs evaluation

In order to determine the study parameters, the costs incurred by the sponsors were first identified, following the pattern of the visits carried out by patients during the observation period. It was complex to determine the costs avoided since each patient may present a different and not easily determinable clinical evolution of IBDs. Simulated therapeutic strategies for IBD patients not assisted by the NHS and referred to our outpatient unit were evaluated in agreement with European Crohn’s and Colitis Organisation (ECCO) guidelines. (Harbord et al., 2017). In order to detect the avoided costs, expenses have been divided into: 1) costs for health services (visits); 2) costs for drugs.

As regards the costs for health services, they were determined through the use of the regional reimbursement instrument called (PAC). For each service provided on an outpatient basis, the Apulia Region reimburses the disbursing body a well-defined sum codified in a reference “list” (see PAC regulation (Deliberazione della giunta regionale 8 marzo, 2021)). As regards the frequency of visits, the one found in normal clinical practice for a patient with moderate-severe IBD was used.

For patients enrolled in clinical trials, the costs for medications and supportive therapies remain the responsibility of the Sponsor. No cost can be charged to the institution or the patient. We estimated the costs for the drugs that the patient would have taken if he had not entered the experimental protocols. In consideration of the inclusion criteria of studies that generally require not to be responders to conventional anti-TNFα therapy, it was estimated that patients would have switched to the second-line biologic drug for IBD therapy, vedolizumab (ENTYVIO) produced by Takeda SA. The frequency of administration was foreseen according to the guidelines (at Time 0, Week 2, Week 6, Week 8, and then every 8 weeks), as recommended by Italian Medicines Agency (AIFA).

### Indirect costs

By “Indirect Costs” we considered the hereby definition: “Indirect costs are facilities and administrative (“F&A”) costs incurred in support of the research infrastructure.” (Clinical Trial Budget Considerations, 2016).

Furthermore, as per the company regulation (ALL1_DDG_266_15.04.2019_DIRGEN.pdf, 2019) on the management of clinical trials: “all costs, even indirect, in the management of clinical trials must be covered by the Sponsor.” For this reason, in addition to the general expenses for the management of the study, specific budget items were provided to cover the indirect costs for: 1) administrative costs for evaluation of the study and “its” activation; 2) expenses for the management of the pharmacy; 3) expenses for the long-term storage of study documents (ISF-Investigator Site File).

These costs were calculated on a flat-rate basis applied to each profit study even if no patient was enrolled.

### Assessment of avoided/averted costs

In this investigation, cost avoidance was defined as “an expenditure that would have been paid to obtain medications but was not spent due to a specific trial-related intervention” as for McDonagh and Lafleur definition (McDonagh et al., 2000), (LaFleur et al., 2004). Similarly to the study conducted by Angerame et al. (2020), for a more thorough evaluation of the avoided costs, along with the savings made for the drugs supplied, although significant, as regards the costs for health services, they were determined through the use of the previously introduced PAC. Indeed, the PAC represents the set of services provided by pathology on an outpatient basis. All the services provided for each visit are aggregated within each PAC and the value is calculated which constitutes the amount paid to the Hospital to compensate the costs incurred. Generally, these costs are lower than what the hospital actually incurs. The costs reimbursed by the Sponsor, on the other hand, fully cover all costs incurred including the items “personnel costs” and general expenses. The aforementioned costs were estimated as the ratio, respectively, between production for the NHS in 1 year (2021) and personnel costs and overheads (i.e., ordinary costs for the NHS, e.g., electricity and heating bills, cleaning and so forth), in the same year. The coefficients obtained were used as an incremental factor in the costs to be borne by the NHS if patients had not entered a clinical trial (see Tables 2, 3). In particular, the analysis calculated the quantities and the price of each drug provided free of charge for the treatment of participants in clinical trials (experimental arm) considered, based on the consideration that the latter trial arm avoided standard treatment, with the assumption of efficacy of the experimental drug at least equivalent to standard therapy.

**TABLE 2:** Overall data collected from sponsor studies.

Studies analysed	11
Pharmacological treatments	217
N of patients	25

**TABLE 3:** Incidence of overheads.

Overheads (in €)	Production for NHS (in €)
5,719,721.19	17,459,168
Ratio	
32.76%	

### Leverage effect estimation

These avoided costs were finally compared with the Direct Investment found in the same studies, thus calculating the leverage effect of the Indirect investment compared to direct investment.

The leverage was calculated as $\frac{\Sigma StudyCosts+\Sigma AvertedCosts}{\Sigma StudyCosts}$ where StudyCosts=PatientCost×N of patients and AvertedCosts=ΣTreatmentCosts+ΣDrugCosts+ΣDirectCosts+ΣHealthcareProfessionalsCosts . All the calculations were conducted in Microsoft Excel 2016 and R version 4.2.0 (2022-04-22) on Windows 11 OS.

## Results

A summary of the collected data was presented in Table 1 and Table 2. The ratio of the incidence of overheads and personnel amounted to 32.76% and 22.69% respectively (see Table 3 and Table 4).

**TABLE 4:** Incidence of personnel.

Personnel expenses (in €)	Production for NHS (in €)
1,304,075.18	17,459,168
Ratio	
22.69%	

The results on the avoided costs for healthcare facilities deriving from the conduct of clinical studies (Table 5), show that, in relation to the sample of five drug companies participating in our 2018-2020 analysis, out of a total of 40,200.42 €, identified as direct investment, 628,158.21 € of avoided costs for the NHS were measured, with an additional saving (leverage effect) for the NHS of 3.67 € for each € invested by the companies promoting clinical trials.

**TABLE 5:** Total direct investments, indirect investments and deriving leverage effect.

Study costs (a) in €	Averted costs (b) in €	Total (c = a+b) in €	Leverage (c/a)
235,102.46	628,158.21	863,260.67	3.67

## Discussion

The main factors that guide the generation of value in clinical research are the quality of the data produced and the time required to obtain it. Even more relevant are the non-economic benefits of clinical research, which make it an undoubted enhancer of public health, connoting it, in this sense, as a public utility: the approximately 35,000 patients directly involved in clinical trials each year benefit from innovative treatments well in advance of their general availability, obtaining early improvements in their condition and quality of life; the hospital and healthcare infrastructures that host clinical research exploit an improvement in the quality of healthcare along a professional growth of the staff involved; the development of new drugs brings social utility, by lengthening the average life span and improving the general quality of life of the population.

The fundamental result of this study on avoided costs is another empirical confirmation, with particular concerns for IBD studies, of the thesis that investments in clinical research have an overall multiplier effect (so-called leverage effect) exploitable by the NHS: indeed for every € invested by the sponsors, it generates an even greater saving, thanks to the drugs provided free of charge to patients enrolled in clinical trials, who otherwise would have to receive a similar therapy at the expense of the NHS.

The simple model exploited found a value of the leverage effect for avoided costs equal to 3.67: This confirms and amplifies the conclusions reached by the previous work on the subject, although in different clinical specialties, of avoided costs (Angerame et al., 2020). This research, on the other hand, confirms for the first time in Italy the data on a sample of 11 gastroenterological studies sponsored by five of the main pharmaceutical companies operating in Italy. It should also be emphasised that what emerged is only a part of the possible benefits for the NHS, given that with regards to indirect investments our study only focused on the savings generated by the supply of drugs, and not on other features such as diagnostic services, hence *de facto* adopting a conservative approach that probably only revealed a part of the total effective savings. This research offers various potentialities of extension and refinement of the results, with the addition of further parameters for a more accurate estimate of the avoided cost and further possible analyses. In terms of possible developments, a further progress to be made would be to define on a wider sample size of clinical studies a more accurate methodology in order to account for a more thorough representation of averted costs, both for the NHS and for the healthcare companies involved in clinical trials.

In conclusion, clinical research in the field of IBDs was confirmed as an engine of economic and social development for a country and its NHS and constitutes a long-term quality investment, which can make an important contribution to recovering from the current health and economic crisis, thanks to the potential for partnerships between the public and private sector.

## Data Availability

The original contributions presented in the study are included in the article/Supplementary Material, further inquiries can be directed to the corresponding author.

## Abbreviations

AIFA, Italian Medicines Agency; CD, Crohn’s Disease; CRO, Clinical Research Organization; ECCO, European Crohn’s and Colitis Organisation; IBD, Inflammatory Bowel Disease; ISCTs, Industry-sponsored clinical-trials; NHS, National Health Service; NIHR, National Institute for Health Research; PAC, Regional reimbursement instrument (in Puglia, Italy); UK, United Kingdom; UC, Ulcerative Colitis; USD, United States Dollars.
